# Letter to the editor: Generation interval for COVID-19 based on symptom onset data

**DOI:** 10.2807/1560-7917.ES.2020.25.29.2001381

**Published:** 2020-07-23

**Authors:** Sergio Bacallado, Qingyuan Zhao, Nianqiao Ju

**Affiliations:** 1 Statistical Laboratory, University of Cambridge, Cambridge, United Kingdom; 2 Department of Statistics, Harvard University, Cambridge, MA, United States

**Keywords:** COVID-19, pre-symptomatic transmission, generation intervals, infection network, markov chain monte carlo

To the editor: The purpose of this letter is to raise a few issues with the methodology of a recent paper by Ganyani et al. [1], which aimed to estimate the parameters of the generation interval of coronavirus disease (COVID-19) using records from Tianjin, China and Singapore at the start of the pandemic.

## Dependence of serial intervals

The likelihood function (in the final displayed equation of the section Methods, Model) suggests that an underlying assumption is that the serial intervals *Z_i_, i* = 2*,...,n* are independent and identically distributed. However, this assumption does not follow from the independence assumptions made in the paper.

Consider a transmission chain *i* → *j* → *k*. Following the authors’ notation, the serial interval between cases *i* and *j* is *Z_j_* = *X_j_* + *Y_j_* where *X_j_* is the generation interval for the transmission *i* → *j* and *Y_j_* = *δ_j_* − *δ_i_* is the difference of the incubation periods of *i* and *j*. Similarly, *Z_k_* = *X_k_* + *Y_k_*. The paper assumes that the generation times *X_j_* and *X_k_* are independent, which may be reasonable. The paper further assumes that generation times and incubation periods are independent, which implies that *X_j_* and *Y_j_* are independent. We argue that the latter assumption is questionable (see below), but let us suppose that it holds. Given these two assumptions, it is clear that the serial intervals *Z_j_* = *X_j_* + *δ_j_* − *δ_i_* and *Z_k_* = *X_k_* + *δ_k_* − *δ_j_* are not independent because the incubation period of case *j* is shared, leading to a *negative* dependence. Similarly, there is a *positive* dependence between *Z_j_* and *Z_k_* in a transmission fork *i* → *j*, *i* → *k*. Hence the likelihood function used in Ganyani et al. [1] may be described as an approximate likelihood.

## Metropolis–Hastings sampler for the infection tree

The Metropolis–Hastings sampler in the script MCMC_generation_interval.R shared with the original article has a proposal distribution which generates trees by sampling a random infector for each non-index case from a list of possible infectors. With the Singapore data, this almost always produces a graph with a cycle, and not a tree, which is inconsistent with the model. Sampling trees consistent with the data uniformly would not be straightforward unless constraints are put on negative generation intervals.

By using a Markov chain Monte Carlo algorithm which augments the state space with the infection time for each case, it is possible to sample the posterior distribution of the infection tree and generation interval parameters using the correct likelihood function. We provide a simple Gibbs sampler which implements this strategy in a Github repository [2].

## Independence of generation interval and incubation period

Consider a transmission *v*(*i*) → *i*. In order to express the density of *Z_i_* as a convolution of *X_i_* and *Y_i_*, a key assumption is that the incubation period *δ_v_*_(_ *_i_*_)_ is independent of the generation time *X_i_* = *t_i_* − *t_v_*_(_ *_i_*_)_. Note that the paper only stated the assumption as “assuming the incubation period is independent of the infection time”, which we interpret as assuming *X_i_* is independent of both *δ_i_* (reasonable) and *δ_v_*_(_ *_i_*_)_ (questionable). The latter independence would clearly not be true if the patient became infectious only after showing symptoms (i.e. *X_i_* ≥ *δ_v_*_(_ *_i_*_)_). Even though the transmission of COVID-19 can be presymptomatic, it still seems difficult to defend the assumption that infectivity is independent of symptom onset biologically.

We believe it is important in further work to test this assumption, or study the sensitivity of the conclusions to deviations from it. Ganyani et al. report rates of pre-symptomatic transmission based on the theoretical model, assuming independence of the generation interval and incubation periods. It would be instructive to, in addition, impute the infection times in the datasets from the posterior distribution, and report the rate of pre-symptomatic transmission among the cases in each dataset.
